# Miglustat in Niemann-Pick disease type C patients: a review

**DOI:** 10.1186/s13023-018-0844-0

**Published:** 2018-08-15

**Authors:** Mercè Pineda, Mark Walterfang, Marc C. Patterson

**Affiliations:** 1 Fundacio Hospital Sant Joan de Déu, Barcelona, Spain; 2 Florey Institute of Neuroscience and Mental Health, Royal Melbourne Hospital, University of Melbourne, Melbourne, Australia; 3 Mayo Clinic, Rochester, MN USA; 4 Hospital Sant Joan de Déu, Passeig de Sant Joan de Déu No. 2, Esplugues, 8950 Barcelona, Spain

**Keywords:** Niemann-Pick disease type C, Miglustat, Efficacy, Biomarker

## Abstract

**Objective:**

Niemann-Pick disease type C (NP-C) is a rare, autosomal recessive, neurodegenerative disease associated with a wide variety of progressive neurological manifestations. Miglustat is indicated for the treatment of progressive neurological manifestations in both adults and children. Since approval in 2009 there has been a vast growth in clinical experience with miglustat. The effectiveness of miglustat has been assessed using a range of measures.

**Methods:**

Comprehensive review of published data from studies of cellular neuropathological markers and structural neurological indices in the brain, clinical impairment/disability, specific clinical neurological manifestations, and patient survival.

**Results:**

Cranial diffusion tensor imaging and magnetic resonance spectroscopy studies have shown reduced levels of choline (a neurodegeneration marker), and choline/N-acetyl aspartate ratio (indicating increased neuronal viability) in the brain during up to 5 years of miglustat therapy, as well as a slowing of reductions in fractional anisotropy (an axonal/myelin integrity marker). A 2-year immunoassay study showed significant reductions in CSF-calbindin during treatment, indicating reduced cerebellar Purkinje cell loss. Magnetic resonance imaging studies have demonstrated a protective effect of miglustat on cerebellar and subcortical structure that correlated with clinical symptom severity. Numerous cohort studies assessing core neurological manifestations (impaired ambulation, manipulation, speech, swallowing, other) using NP-C disability scales indicate neurological stabilization over 2–8 years, with a trend for greater benefits in patients with older (non-infantile) age at neurological onset. A randomized controlled trial and several cohort studies have reported improvements or stabilization of saccadic eye movements during 1–5 years of therapy. Swallowing was also shown to improve/remain stable during the randomized trial (up to 2 years), as well as in long-term observational cohorts (up to 6 years). A meta-analysis of dysphagia – a potent risk factor for aspiration pneumonia and premature death in NP-C – demonstrated a survival benefit with miglustat due to improved/stabilized swallowing function.

**Conclusions:**

The effects of miglustat on neurological NP-C manifestations has been assessed using a range of approaches, with benefits ranging from cellular changes in the brain through to visible clinical improvements and improved survival.

**Electronic supplementary material:**

The online version of this article (10.1186/s13023-018-0844-0) contains supplementary material, which is available to authorized users.

## Background

Niemann-Pick disease Type C (NP-C) is a rare neurovisceral lysosomal disorder caused by autosomal recessive mutations in either the *NPC1* gene (in 95% of cases) or the *NPC2* gene (in approximately 4% of cases) [1–3]. NP-C is panethnic, and is usually sporadic, but specific clinical sub-populations are associated with a higher risk of NP-C compared with the general population [4]. Widely recognised age-at-onset categories are as follows: perinatal (onset at age < 3 months, including prenatal onset); early-infantile (at age 3 months to < 2 years); late-infantile (at age 2 to < 6 years); juvenile (at age 6–15 years); and adolescent/adult (at age > 15 years) [5, 6]. While NP-C is traditionally recognised as a childhood-onset disease, a greater proportion of adolescent/adult-onset cases are now being detected. In particular, patients with movement disorders, organic psychosis, or early-onset cognitive decline are being diagnosed [4, 7–9]. This trend is expected to continue, partly owing to raised awareness of the disease [10, 11], as well as the increasing availability of rapid, convenient diagnostic biomarkers [12–15], improved genetic analytic methods [7, 16, 17], and new clinical screening tools [17–20].

As in many rare inherited metabolic diseases, the clinical presentation of NP-C is highly heterogeneous [5, 21]. The signs and symptoms of NP-C can be broadly grouped into three categories: visceral, neurological and psychiatric. Patients with the perinatal and early-infantile forms tend to present with visceral symptoms, cholestasis and development delay, while those with juvenile- and adolescent/adult-onset forms may present with a wide range of neurological manifestations [1, 22]. Typical neurological manifestations include cerebellar ataxia, dysmetria, dysarthria, and dysphagia [5, 23, 24]. Vertical supranuclear saccade palsy (VSSP), and gelastic cataplexy are characteristic neurological signs [5]. In addition, patients with adult-onset disease often present with neuropsychiatric signs including early cognitive decline and/or psychiatric disturbances [25–28].

There is no cure for NP-C, although research into possible disease-modifying therapies has been ongoing since the 1950s [29]. Unesterified cholesterol was originally considered the key offending metabolite underlying the biochemical defect. The therapeutic effect of lipid-lowering agents was therefore investigated [30, 31]. While these agents reduced hepatic and plasma cholesterol levels, no effect on neurological progression of the disease was seen. A number of other potential therapies have been investigated, but so far only limited, mainly experimental data have been reported [5]. Until fairly recently, treatment for NP-C has therefore been restricted to palliative approaches aimed at ameliorating neurological manifestations. These generally include anti-seizure drugs, anticholinergics to relieve dystonia and tremor, and antidepressants or antipsychotics for mood and psychotic disorders [5, 6].

Impaired intracellular lipid trafficking and resultant accumulation of a broad range of lipids in cell lysosomes and late endosomes is considered the primary underlying pathophysiological defect in NP-C [32, 33]. Current understanding of the link between impaired lipid storage and subsequent neurodegeneration remains incomplete as the exact functions of *NPC1* and *NPC2* proteins are not fully known, and different tissues show different patterns of lipid accumulation [34]. Miglustat (Zavesca™; Actelion Pharmaceuticals Ltd) inhibits the synthesis of glycosphingolipids (GSLs) and is the first and only targeted therapy to be approved for the treatment of NP-C [5, 35, 36]. As required for any treatment for diseases associated with centrally-mediated neurological symptoms, miglustat is able to cross the blood–brain barrier, which allows it to access malfunctioning neurons in the brain [37].

In animal models of NP-C, miglustat has been shown to reduce neuronal glycosphingolipid accumulation, delay the onset of neurological dysfunction, and prolong survival [38, 39]. A clinical proof-of-concept study showed improved lipid trafficking in peripheral blood B lymphocytes after miglustat treatment [40]. Subsequently, a randomised controlled trial (RCT), long-term extension studies, and a retrospective cohort study formed the clinical evidence base for initial regulatory approval in the EU in 2009: miglustat is now available in many countries worldwide [41–43]. While a key effect of miglustat on glycosphingolipid metabolism has been suggested [35], the precise mode of action of this agent is not yet fully understood.

There has been a vast growth of clinical experience with miglustat in treating patients with NP-C since it first became available, which has prompted development of a variety of methods to measure disease responses to therapy. Most commonly, the clinical impact of miglustat on observable, core neurological manifestations has been evaluated using subjective physician-reported assessments of patient disability [24, 42, 44–48]. However, such NP-C disability scales and ambulation indices are not easy to apply in patients with early- or late-infantile onset. Clinical developmental scales such as the Denver Developmental Screening Test (DDST [49]) and the Bayley scales for infant development (Bayley-III [50]) can be helpful for assessing patients during infancy.

Objective, quantitative assessments of specific clinical features such as ocular motor function and swallowing impairment can allow tracking of neurological progression independent of patient age [51–56]. A number of studies have also reported objective parameters for measuring changes in brain neurochemistry, structure, and neuronal transmission [57–59]. No standardised disease-specific approaches for assessing psychiatric manifestations in NP-C have been reported, although existing clinical scales can be used to assess cognitive function [59–61].

This paper provides a comprehensive review of published data from studies that have reported therapeutic effects with miglustat in patients with NP-C. Given the multi-faceted nature of NP-C, we focus on the rationale and clinical utility of methods used to evaluate a range of different aspects of the disease, covering both subjective and objective efficacy measures. This review is intended to provide guidance for the choice of disease response markers for future evaluations of miglustat efficacy. A full consensus recommendation on the best methods for assessing the effectiveness of targeted therapy would be a desirable endpoint in NP-C. However, in lieu of sufficient data to meet this goal, this article serves as a source of information for future studies.

## Miglustat pharmacodynamics and pharmacokinetics in NP-C

Miglustat is a small iminosugar molecule that reversibly inhibits glucosylceramide synthase – the enzyme that catalyses the first committed step in the GSL synthesis pathway [35]. This activity has been demonstrated to ameliorate the lipid-trafficking defect in patients with NP-C, reducing intracellular lipid storage and normalizing lipid transport in peripheral blood B-lymphocytes [40]. In turn, this is thought to reduce potentially neurotoxic levels of gangliosides GM2 and GM3, lactosylceramide and glucosylceramide.

Data from animal studies suggest that miglustat can delay the progression of NP-C and prolong survival [39, 62]. Miglustat was shown to reduce cerebellar pathology and storage of GM2 and GM3 gangliosides, to decrease the risk of neurological manifestations developing by approximately half, and to increase lifespan in NP-C mice after approximately 1 year of treatment [39]. In a feline model miglustat decreased brain ganglioside accumulation and delayed the onset and progression of neurological symptoms [39]. Miglustat has also been shown to improve Purkinje cell survival in cats, possibly related to modulation of microglial immunophenotype and function [63].

Data from in vitro and ex vivo analyses in animals and humans indicate that miglustat may modulate intracellular calcium homeostasis through its effects on glucosylceramide levels [64]. Impaired calcium homeostasis related to excess sphingosine storage is a suspected initiating factor in the pathogenesis of NP-C [65, 66]. Lysosomal sphingosine accumulation in *NPC1*-mutant cells is thought to inhibit lysosomal calcium uptake [65], which in turn may lead to impaired endocytic function and subsequent development of the NP-C disease phenotype. Miglustat may improve intracellular calcium balance by reducing sphingosine accumulation [65]. Recent findings of reduced calcium binding protein levels in cerebellar neurones suggest a role for calcium homeostasis in the therapeutic effects of miglustat [67].

Pharmacokinetic studies in humans have shown that miglustat is rapidly absorbed after oral administration, reaching maximal plasma drug concentration in 2–2.5 h [36, 68]. The absolute bioavailability of miglustat is at least 80%, and its pharmacokinetic profile is approximately dose-proportional [36]. The large volume of distribution of miglustat (83–105 L) reflects the fact that it is not restricted to the bloodstream after absorption, having physico-chemical properties that enable wide distribution in extravascular tissues as well as its ability to cross the blood–brain barrier [36–38]. Miglustat is not metabolized in vivo and is eliminated mainly via the kidneys [36]. With a terminal elimination half-life (t_½_) of 6–7 h, steady-state pharmacokinetic conditions are achieved 4–6 weeks of after initiation of treatment [69].

## Clinical efficacy studies

A wide range of measures have been developed and employed to assess the effects of miglustat therapy over the last decade. The sections below review data on the effectiveness of miglustat from three categories of published reports: 1) studies/case series in paediatric patients (Table 1); 2) adult and across-ages cohort studies (Table 2); and 3) key single-patient case reports (Table 3). Findings are discussed in sequence according to the type of disease features they address, including: neuropathological markers; specific neurological and psychiatric manifestations; general clinical impairment/disability; and treatment outcomes (survival).

**Table 1:** Summary of studies/case series in paediatric patients

Study / ref	Design	Pts / controls (N)	Mean (range) pt age^a^	Biomarkers and surrogate biomarkers	Median (range) treatment duration	Reported treatment effects
Patterson et al. (2010) [41]	12-mo prospective multicentre Phase II RCT, 12-mo extension and continued extension	LI, JUV pts. (*N* = 12)	7 (4–11) yrs	• HSEM-α and HSEM-β • Swallowing • SAI	2.9 (2–4) yrs	• Stabilised HSEM, swallowing, SAI • Stabilisation in 80% pts. at 24 mo
Pineda et al. (2010) [45]	Paediatric multicentre case series with up to 52-mo follow up	EI, LI, JUV pts. (*N* = 16)	NR (1–15) yrs	• Disability scale • Cog. function (DDST, WISCR) • Cranial imaging (PET) • Biomarkers (ChT, CCL18)	NR (0.5–4) yrs	• Stabilised disability scores • Stable cerebral hypometabolism • Generally stable ChT and CCL18 • Greatest benefits in older pts.
Héron et al. (2012) [44]	Paediatric prospective open-label multicentre observational study	EI, LI, JUV pts. (*N* = 20)	3 (< 1–7) yrs	• Disability scale • MRI • MRS	EI: 1.3 (NR) yrs LI: 1.0 (NR) yrs JUV: 1.0 (NR) yrs	• Neurological improvement/stabilisation more frequent in LI and JUV vs. EI pts
Fecarotta et al. (2011) [51]	Prospective open-label single-centre observational study	EI and JUV pts. (*N* = 4)	NR (< 1–11) yrs	• VFS • Disability scale	NR (3.0–4.0) yrs	• Improved swallowing function • Decreased aspiration
Karimzadeh et al. (2013) [47]	Paediatric multicentre case series with up to 26-mo follow up	EI, LI, JUV pts. (*N* = 21)	NR (< 1–11) yrs	• Disability scale • Psychomotor development	EI: 0.8 (NR) yrs LI: 1.2 (NR) yrs JUV: 1.3 (NR) yrs	• Ambulation, fine/gross motor movements, swallowing, speech and VSSP generally stabilised • Reduced psychomotor delay

^a^Age at disease onset or diagnosis; *ChT* chitotriosidase, *DDST* Denver developmental screening test, *EI* early-infantile, *HSEM* horizontal eye movements, *JUV* juvenile, *LI* late infantile, *MRI* magnetic resonance imaging, *MRS* magnetic resonance spectroscopy, *NR* not reported, *PET* positron emission tomography, *pt./pts.* patient(s), *RCT* randomised controlled trial, *SAI* standard ambulation index, *VFS* video-fluoroscopic analysis, *WISCR* Wechsler Intelligence Scale for Children

**Table 2:** Summary of adult and across-ages cohorts

Study / ref	Design	Pts / controls (N)	Mean (range) pt age^a^	Biomarkers and surrogate biomarkers	Median (range) treatment duration	Reported treatment effects
Patterson et al. (2007) [56]	12-mo prospective multicentre Phase II RCT	JUV and A/A pts. (*N* = 29)	Miglustat pts. 25 (12–42) yrs. Std care: 23 (13–32) yrs	• HSEM-α/HSEM-β • Swallowing • SAI • Hearing	*Adol/adults* 1 (0.5–1.2) yr *Children* 1 (0.2–1.1) yr	• Stabilised SEM, swallowing, SAI • Disease progression stable in 80% pts
Pineda et al. (2009) [42]	Retrospective multicentre observational study	EI, LI, JUV, A/A pts. (*N* = 66)	10 (0–32) yrs	• NP-C disability scale • Disability score	1.5 (0.1–4.5) yrs	• Stabilised disability scores • Stabilised cerebral hypometabolism • Greater benefits in LI and JUV vs. EI pts
Wraith et al. (2009) [114]	Post hoc analysis from Phase II RCT	LI, JUV, A/A pts. (*N* = 29)	A/A: 23 (NR) yrs LI/JUV: 7 (NR) yrs	• Disease stability	1.0 (NR) yr	• Stabilisation in 21/29 (72%) pts. overall • Stabilisation in 8/10 (80%) children
Galanaud et al. (2009) [79]	Single-centre case series with 24-mo follow up	A/A pts. (*N* = 3)	17 (16–19) yrs	• MRI • MRS	2.0 (NR) yrs	• Clinical improvement or stabilization • Sustained improvement in Cho/Cr ratio
Wraith et al. (2010) [43]	12-mo open-label extension and continued extension of Phase II RCT	A/A pts. (*N* = 21)	25 (12–42) yrs	• HSEM-α • Swallowing • SAI • Cog. function (MMSE)	1.9 (0.1–2.0) yrs	• Stabilised HSEM-α, ambulation and swallowing
Walterfang et al. (2012) [121]	Systematic literature review and longitudinal meta-analysis	EI, LI, JUV and A/A pts. (*N* = 97)	NR	• Survival • Dysphagia	NR	• Potential survival benefit • Survival related to reduced dysphagia
Fecarotta et al. (2015) [48]	Prospective open-label multicentre observational study	EI, LI, JUV, A/A pts. (*N* = 25)	13 (< 1–44) yrs	• Disability scale • Clinical swallowing studies • Developmental delay • Cog. impairment	5.9 (4.0–8.0) yrs	• Stabilised/improved neurological manifestations • Greater benefits with earlier treatment
Bowman et al. (2015) [57]	Prospective open-label single-centre observational study	A/A pts. vs. healthy controls (*N* = 26)	28 (14–47) yrs	• MRI • HSEM-α, HSG • Disability scale • Ataxia (BARS)	2.8 (NR) yrs	• Protective effect on cerebellar Purkinje neurones • Benefits in key brain regions
Patterson et al. (2015) [46]	Registry-based multicentre observational study	EI, LI, JUV, A/A pts. (*N* = 92)	10 (< 1–45) yrs	• Disability scale	3.9^b^ (1.1–9.8) yrs	• Reduced disease progression • Greater benefits in older pts
Sedel et al. (2016) [80]	Prospective open-label multicentre observational study	JUV and A/A pts. (*N* = 16)	17 (9–32) yrs	• Disability scale • MRS	1.6 (0.5–8) yrs	• Improved Cho/NAA ratio • Reduced disease progression
Lau et al. (2016) [88]	Prospective, open-label single-centre observational study	EI, LI, JUV, A/A pts. (*N* = 39)	11 (1–22) yrs	• NNSS • DTI	NR	• Lower NNSS severity scores across a range of cerebellar DTI measures
Bradbury et al. (2016) [67]	Prospective open-label single-centre observational study	EI, LI, JUV, A/A pts. (*N* = 36)	11 (2–51) yrs	• CSF calbindin • NNSS	NR (0.5–1.25) yrs	• Reduced CSF calbindin vs. controls
Masingue et al. (2017) [58]	Retrospective open-label single-centre observational study	A/A pts. vs. healthy controls (*N* = 26)	MRI: 18 (5–56) yrs DTI: 16 (5–30) yrs	• Disability scale • MRI • DTI	5.0^b^ (1.0–9.0) yrs	• Improved FA in some brain regions • Clinical and MRI metrics not correlated
Heitz et al. (2017) [111]	Retrospective open-label single-centre observational study	A/A pts. (*N* = 21)	35 (NR) yrs	• Cog. function (MMSE/FAB) • Disability scale	1.5 (NR) yrs	• Stable neuropsychological scores

^a^Age at disease onset or diagnosis; ^b^mean duration; *A/A* adolescent/adult onset, *BARS* Brief ataxia rating scale, *Cho* choline, *ChT* chitotriosidase, *Cr* creatine, *CSF* cerebrospinal fluid, *DTI* diffusion tension imaging, *EI* early-infantile, *FA* fractional anisotropy, *FAB* frontal assessment battery, *HSEM* horizontal saccadic eye movements, *HSG* horizontal saccadic gain, *JUV* juvenile, *LI* late infantile, *MMSE* mini-mental state examination, *MRI* magnetic resonance imaging, *MRS* magnetic resonance spectroscopy, *NAA* N-acetyl aspartate, *NNSS* NIH neurological severity scale, *NR* not reported, *pt./pts.* patient(s), *RCT* randomised controlled trial, *SAI* standard ambulation index

**Table 3:** Summary of key case reports

Study / ref	Design	Pts / controls (N)	Mean (range) pt age^a^	Biomarkers and surrogate biomarkers	Median (range) treatment duration^b^	Reported treatment effects
Chien et al. (2007) [53]	Case report on 2 pts. with 1-yr follow up	EI, JUV pts. (*N* = 2)	–	• VFS • HSEM-α, visual pursuit • Cog. function (MMSE)	1 yr	• Improved/stabilised swallowing and ambulation • Stable plasma ChT
Santos et al. (2008) [109]	Single-pt. case report with 12-mo follow up	JUV pt. (*N* = 1)	–	• Disability scale • Psychology and behaviour (CBCL)	1 yr	• Stable disability scores • Improved speech, movement and seizures • Improved cog. Function • Reduced depression/affective symptoms
Scheel et al. (2010) [87]	Single-pt. case report with 12-mo follow up	A/A pt. vs. age-matched controls (*N* = 18)	29 yrs	• DTI • Saccadic eye movements	1 yr	• Improved FA in the corpus callosum
Di Rocco et al. (2012) [124]	Case report on 2 pts. with up to 7 yrs’ follow up	EI pts. (*N* = 2)	Pt 1: 7 mo Pt 2: 19 mo	• Neurological examination • Mental development scale • MRI	Pt 1: 5 yrs Pt 2: 7 yrs	• Neurological stabilisation
Chien et al. (2013) [52]	Single-centre case series with up to 6-yr follow up	EI, JUV pts. (*N* = 5)	NR (4–8) yrs	• VFS • Cog. function (MMSE) • SAI	4 (4–6) yrs	• Swallowing function stabilised • Improved/stabilised cognitive function • 2-year stabilisation of ambulation
Mattsson et al. (2013) [110]	Single-pt. case report	A/A pt. (*N* = 1)	44 yrs	• Neurological examination • Ocular motor examination • MRI, SPECT, EEG	NR	• Improved speech (from mutism to complete, coherent sentences)
Szakszon et al. (2014) [102]	Single-pt. case report with 3-yr follow up	JUV pt. (*N* = 1)	10 yrs	• Neurological examination • Psychiatric consultation • MRI	3 yrs	• Complete recovery from psychosis
Maubert et al. (2015) [107]	Case report in sibling pair	A/A pt. (*N* = 1)	Pt. 1: 22 yrs Pt. 2: 21 yrs	• Neurological examination • Cog. function (MMSE)	Pt 1: 1.7 yr Pt2: NA	• Stable psych. Symptoms and cog. Function • Cessation of anti-psychotic therapy • Greater independence
Cuisset et al. (2016) [99]	Single-pt. case report in atypical NP-C	JUV pt. (*N* = 1)	10 yrs	• Swallowing (VFS) • SAI	3 yrs	• Global improvement • Improved cog. and ambulatory function • Abolition of seizure activity
Abe and Sakai (2017) [97]	Single-pt. case report with 20-yr follow up	JUV pt. (*N* = 1)	20 yrs	• Neurological examination • Swallowing (clinical) • MRI	4 yrs	• Improved swallowing capacity • Improved and muscle tone
Benussi et al. (2017) [59]	Prospective, single-centre case-control study	A/A pts. vs. healthy controls (*N* = 22)	35 (25–57) yrs	• TMS • Cog. function (MMSE, other)	1.0 (NA) yr	• Improved LTP-like cortical plasticity • Improved short-latency afferent inhibition • Improved/stabilised movement
Hassan et al. (2017) [93]	Single-pt. case report in a pt. with cerebellar ataxia	A/A pt. (*N* = 1)	–	• TMS • Cog. function	1.3 yrs	• Reduced short-latency afferent inhibition (sensorimotor integration)

^a^Age at disease onset or diagnosis; ^b^single-patient follow up for case reports; *A/A* adolescent/adult-onset, *CBCL* Child behaviour checklist, *ChT* chitotriosidase, *DTI* diffusion tension imaging, *EEG* electroencephalography, *EI* early-infantile, *FA* fractional anisotropy, *HSEM* horizontal saccadic eye movements, *JUV* juvenile, *LI* late infantile, *MMSE* mini-mental state examination, *MRI* magnetic resonance imaging, *NR* not reported, *SAI*, standard ambulation index, *TMS* transcranial magnetic stimulation, *VFS* video-fluoroscopic analysis

### Neuropathological markers

Excess GM2 and GM3 gangliosides and unesterified cholesterol are differentially stored in neurons of the cerebral cortex, cerebellum, and hippocampus in NP-C. This results in a range of recognisable ultrastructural changes in these brain regions, including ballooned neurones, meganeurites, and glial cell abnormalities [62, 70, 71]. Neurodegeneration is most dramatic in the cerebellum, where Purkinje cell death is a hallmark neuropathological feature of NP-C [70, 72]. Cerebellar Purkinje cells are particularly sensitive to GM2 and GM3 ganglioside accumulation, but the basis of this selective vulnerability is not yet known [73, 74]. Alzheimer disease (AD)-like features including amyloid beta-protein accumulation – without plaque formation – and paired-helical filament neurofibrillary tangles (NFTs) are consistently seen in NP-C, mostly in the basal ganglia, hypothalamus, brain stem and spinal cord [75, 76], and are possibly related to dysregulation of cholesterol metabolism and apolipoprotein E genotype [76, 77]. While many of these features are only observed on post-mortem histological examination, some of them, in particular cerebellar Purkinje cell degeneration, bear relevance to clinical disease monitoring methods.

#### Brain imaging markers

Objective clinical markers of some of the known neuropathological features of NP-C have been reported based on serial, quantitative brain imaging measurements in longitudinal clinical studies. Importantly, the required technologies for most assessments are available in many hospitals. However, some of these techniques are not suitable for clinical practice settings, requiring specialist imaging analysis post-processing and expertise.

Brain choline (Cho), creatine (Cr) and *N*-acetylaspartate (NAA), and derivative Cho/Cr and Cho/NAA ratios are acknowledged markers for neurodegeneration in the brain, and are detectable using brain magnetic resonance spectroscopy (MRS) [78]. In a case series of three French adolescent/adult-onset NP-C patients, Galanaud et al. reported a sustained decrease in Cho/Cr ratio during 2 years of miglustat therapy [79]. These changes were associated with mild improvement or stabilization of core neurological manifestations, as measured using an NP-C disability scale [24]. Improved neuronal viability (based on Cho/NAA ratios) was later reported in a larger cohort of 16 adolescent/adult patients who received miglustat for an average of 2.5 years. In contrast, patients who discontinued miglustat showed deterioration [80]. In line with MRS findings, annual progression of NP-C disability scores improved in patients who continued miglustat, but worsened in those who discontinued therapy.

Positron emission tomography (PET) has also been used to assess brain metabolism in patients with NP-C [45, 81]. Serial PET imaging was used to monitor treatment response in the frontal and temporo-parietal regions, basal ganglia, and cerebellum in a case series following 16 Spanish paediatric NP-C patients [45]. Cerebral hypometabolism appeared stabilized and neurological disease progression slowed during 3–4 years of miglustat treatment in patients with juvenile-onset disease. However, PET data were less consistent in patients with early-infantile or late-infantile onset forms. Overall, no clear relation between PET findings and NP-C disability scores was observed. Further studies are required to assess the utility of PET in disease monitoring, particularly in the adolescent/adult-onset form.

Cerebral atrophy involving both grey- and white-matter regions is frequently seen on clinical imaging in NP-C, and some of these changes correlate with clinical functional deficits. For instance, MRI volumetry indicates that reduced area and thickness of the corpus callosum, decreased cerebellar volume, decreased volume of subcortical nuclei (including the thalamus, basal ganglia and hippocampus), and ratio of pontine-to-midbrain area correlate with saccadic eye movement indices and loss of motor function (e.g., ataxia, manipulation) [54, 55, 82–84].

In a prospective, controlled study, the loss of cerebellar grey and white matter, bilateral thalamic volume, and right caudate volume was slower in adolescent/adult-onset NP-C patients who received miglustat for a median period of 2.8 years than in untreated patients [57]. Loss of cerebellar grey matter and left thalamic volumes correlated with NP-C disability scores and decreased horizontal saccadic gain (HSG), and a potential protective effect of miglustat on cerebellar Purkinje neurons was proposed [57].

Diffusion tensor imaging (DTI) is another neuroimaging method that has been used in NP-C to measure white matter architecture and integrity, and may be useful for identifying regional changes in myelination and axonal integrity, which are known to be altered in NP-C [83, 85, 86]. A case report using DTI in an adult NP-C patient showed improved fractional anisotropy (FA), which is a marker of axonal myelin integrity, in the corpus callosum after 1 year of miglustat therapy [87]. In a prospective longitudinal cohort study in 39 NP-C patients, miglustat therapy was associated with lower neurological symptom severity scores and less pathological change across a range of cerebellar DTI measures (FA, mean diffusivity and cerebellar regional volumes) [88]. Serial DTI assessments in a cohort of 13 adolescent/adult-onset patients showed improved FA in key white matter regions including the corpus callosum, forceps minor and the cingulate gyrus after 2 years of miglustat therapy [58]. Decreased (improved) radial diffusivity (RD) was also noted in the corpus callosum. These beneficial changes were considered possibly related to effects on cerebral metabolism. A case-control DTI study comparing seven miglustat-treated and two untreated control patients showed that miglustat treatment was associated with slowed degeneration in the corticospinal tract, thalamic radiation and inferior longitudinal fasciculus (Bowman et al., JIMD in press) [89].

#### CSF markers

Bradbury, et al. reported changes in cerebrospinal fluid (CSF) levels of the calcium-binding protein, calbindin D-28 K [67]. This putative marker is present at high levels in the dendrites, soma and axons of cerebellar Purkinje cells, and elevated CSF calbindin is considered a marker for Purkinje cell loss [67]. Prospective analyses in 36 patients with NP-C aged 1.8–51.3 years indicated significant (approximately 33%) decreases in CSF calbindin during 2 years of miglustat therapy. However, unlike MRS-derived indices described above, CSF calbindin did not appear to correlate with neurological severity scores, as measured using the NIH NP-C neurological severity scale described by Yanjanin et al. [90].

Cologna et al. [91] reported altered levels of the CSF fatty acid binding protein, FABP3, among other protein biomarkers (e.g., oxidative stress proteins glutathione s-transferase alpha, superoxide dismutase) in patients with NPC1 in a study assessing the potential underlying pathophysiology of NP-C. Furthermore, levels of FABP3, which is highly expressed in cerebellar Purkinje cells and is considered a marker of ongoing neuronal cell loss/damage, were significantly decreased in miglustat-treated patients relative to untreated patients.

#### Electrophysiological markers

Neurophysiological indices derived from transcranial magnetic stimulation (TMS) tests have been suggested as possible quantitative biomarkers for disease progression [59]. TMS testing in a preliminary study of two patients with NP-C showed improvement after 1 year of miglustat treatment, and impaired SAI scores appeared to correlate with disease severity and *NPC1* mutation status, as supported by previous evidence [92]. However, the control population for this study was not well matched, and these findings should be interpreted with caution. In a further case report relating to longitudinal TMS evaluations during 15 months of miglustat therapy, cerebellar inhibition, TMS-derived short-latency afferent inhibition – a measure for cholinergic transmission – and short-interval intracortical facilitation – a marker for glutamatergic neurotransmission – both showed improvement during miglustat therapy [93].

### Assessments of key clinical neurological manifestations

#### Ocular motor assessments

Ocular-motor abnormalities, which often precede other motor symptoms, are a hallmark feature of NP-C and have been reported in 65–81% of patients [5, 23, 27]. This variability on reported rates likely reflects under-ascertainment of a finding that is frequently overlooked. In the experience of the authors, VSSP is invariably present in patients with neurologic symptoms beyond infancy. Voluntary vertical saccades are affected first, usually in the late-infantile period, and are followed over time by reduced pursuit movements and impaired horizontal saccades [82, 94]. Full VSSP eventually develops in most patients, reflecting progressive degeneration of ocular-motor nerve tracts in the brainstem [82, 84]. Vertical saccades are often heavily impacted, if not absent, by the time a patient has been diagnosed with NP-C, which prevents their effective use in quantifying disease progression beyond diagnosis. In contrast, horizontal saccades deteriorate more gradually as the disease progresses [95, 96]. Horizontal saccadic eye movement parameters are therefore more useful for measuring miglustat treatment effects.

Between two indices of the linear relationship between peak horizontal saccade duration and amplitude, horizontal SEM alpha (HSEM-α; the gradient) has been verified as a useful marker for neurological progression and treatment effects in NP-C, while HSEM-β (the intercept) is considered less relevant [54, 56]. In addition, a number of other saccadic eye movement measures that reflect volitional or even cognitive aspects can also be used to indicate neurodegeneration in different regions of the brain (e.g., corpus callosum, parietal cortex, cerebellar vermis, frontal cortex, and basal ganglia projections to the brainstem) [54, 55, 84].

HSEM-α (ms/deg) was the primary efficacy endpoint in the seminal 12-month RCT investigating treatment with miglustat versus standard care in 41 adults and children with NP-C [56]. After 12 months of treatment, HSEM-α was improved in miglustat-treated adults compared with those who received standard care. This effect was statistically significant after exclusion of patients taking benzodiazepines, which are known to impair saccadic eye movements. A mean improvement in HSEM-α was also observed in children. In both adults and children who completed 12 months of miglustat therapy and subsequently participated in open-label extension treatment, HSEM-α was maintained/stable relative to baseline up to 24 months and beyond [41, 43]. While HSEM-β indicated increases (deterioration) in patients aged ≥12 years or older, smaller changes were seen in miglustat-treated patients compared with those on standard care. Changes over 12 months were not statistically significant (*p* = 0.834).

Changes in a number of saccadic eye movement parameters were reported in an observational cohort study in nine adolescent/adult NP-C patients who received miglustat for up to 5 years [54]. HSG, a strong index of cerebellar vermian integrity, and self-paced saccades, which reflect function of frontal-lobe eye fields in the brain, were reported to discriminate better than HSEM-α between NP-C patients and controls, and as potentially more robust indicators of treatment efficacy. Both parameters were significantly improved in treated patients but worsened significantly in untreated patients. HSG was also measured in the MRI study in adolescent/adult patients reported by Bowman et al. [57]. Correlations between the rates of change in HSG and disease-relevant cerebellar grey and white matter regions were observed. While HSG increased in treated patients during a median 2.8 years of miglustat therapy, it decreased in untreated patients. This treatment difference was highly statistically significant.

Detailed, standardised ocular motor assessments are crucial in clinical practice for the diagnosis/detection of NP-C [5]. As well as saccades, smooth pursuit, gaze-holding function, optokinetic nystagmus, and vergence movements should be examined (http://www.neurocular.com) [16]. A number of studies have also clearly demonstrated that saccadic eye movement measurements are useful for assessing patient responses to therapy in NP-C [41, 43, 54, 56, 57]. Video-oculography (VOG; e.g., EyeSeeCam) allows the semi-automated recording of all types of eye movements, and can be used for objective, quantitative analysis of ocular motor function (http://eyeseecam.com/) [59].

#### Swallowing

Dysphagia is a very common and frequently progressive manifestation of NP-C, and is reported in approximately 80% of patients [21–23]. Similar to dysarthria, dysphagia arises in NP-C chiefly through neurodegeneration in specific brain regions including the corticobulbar pathways, basal nuclei, brainstem and cerebellum. Patients with impaired swallowing need to be closely monitored to avoid pneumonia subsequent to food aspiration. Dysphagia has been studied in NP-C using both subjective clinical assessments based on clinical scales, and objective, semi-automated instrumental methods that directly assess swallowing function.

Standardised clinical assessments of patients’ ability to swallow different foods, graded using a five-point categorical scale, were performed during the 12-month RCT and long-term extension study of miglustat in adults and children [41, 43, 56]. Improved/stable swallowing was reported in the majority of juvenile- and adolescent/adult-onset patients after 12 and 24 months of miglustat therapy (86% and 79–93%, respectively) [43, 56]. While swallowing difficulties were less common among children compared with older-onset patients, likely due to limited time for neurological deterioration to become apparent, no deterioration in swallowing function was seen in children after 24 months on therapy [41]. Similar swallowing assessments were employed in a cohort study including 25 NP-C patients (all age groups), and demonstrated improved/stabilized swallowing function in the majority of patients (65%) treated with miglustat for 24 months [48]. These results persisted in 40–50% of patients after 48–96 months.

Dysphagia is one of the cardinal neurological manifestations evaluated by clinical NP-C disability scales, where swallowing function is generally scored across severity ratings from ‘normal’ (lowest score) through to ‘requirement for nasogastric tube or gastric button feeding’ (most severe score). In an observational retrospective study in 66 patients across all age-at-onset categories (range 0–32 years), stable/improved scores on the dysphagia subscale were observed in 81% of patients after a median of 1.5 (0.1–4.5) years on miglustat [42]. A comparable proportion (75%) of patients included in the international NPC Registry also showed stable or improved swallowing function based on disability scores [46].

Silent aspiration (i.e., food or fluid aspiration without overt, observable signs of choking) of small or trace amounts of food or fluid is not well diagnosed without objective, quantitative data derived from video-fluoroscopic (VFS) analyses. Chien et al. reported VFS findings in two Taiwanese patients treated with miglustat for 1 year [53]. Patient 1 had severely impaired swallowing at baseline and showed substantial improvements by month 6 of treatment. Patient 2 had a later disease onset but showed impaired cognition that improved during miglustat therapy: his swallowing ability was normal at baseline and remained stable throughout treatment. In a later case series, the same research group reported improved or stable VFS-defined swallowing function across five patients who received miglustat for a median of 4 years in a Taiwanese case series [52]. No significant increases in the Han dysphagia scale or an aspiration–penetration index were observed among four evaluable patients.

VFS studies in an Italian case series of four early-infantile and juvenile-onset patients with NP-C who received miglustat for 3–4 years indicated early improvements in swallowing ability on treatment [51]. Importantly, VFS studies in the Italian patients indicated that more severe, pharyngeal-phase swallowing impairments associated with penetration/aspiration of VFS contrast agent occurred later in the course of disease [51]. This finding was in line with parallel ratings of overall neurological impairment. Further, therapeutic effects on pharyngeal-phase swallowing appeared earlier and were more pronounced than those on oral-phase swallowing, which hints at a selective effect on involuntary reflexes dependent on the integrity of brainstem neurones. More recently, Abe and Sakai reported improved swallowing in a single-patient case report [97].

#### Ambulation

Cerebellar ataxia and dystonia are core neurological manifestations in NP-C [4, 22]. The treatment effects of miglustat on ambulation are usually assessed based on subjective clinical observation and findings from neurological examinations in clinical practice settings [23, 27, 28]. However, ambulation has been studied using more structured assessments such as the SAI, where it is rated on a categorical scale from zero (asymptomatic or fully active) to 9 (restricted to wheelchair and unable to transfer independently) [98].

SAI assessments during the 12-month miglustat RCT and subsequent extension treatment demonstrated stabilised ambulation in both adults and children over 24 months of therapy [41, 43, 56]. In the Taiwanese paediatric case series reported by Chien et al., SAI-defined ambulatory function remained stable for at least the first 2 years of treatment in most patients, but there was a trend towards deterioration thereafter, considered possibly related to treatment interruptions [52, 53]. Substantial improvements, concomitant with improved cognition and abolition of seizure activity, have also been reported using the SAI in a French patient with juvenile-onset NP-C [99].

Ambulation subscales are consistently included in NP-C disability scales, which are most often based on the original Iturriaga scale or a modified version thereof [24, 45]. Longitudinal analysis of disability assessments in 92 patients across all age-at-onset categories (range < 1–44.6 years) included in the international NPC Registry indicated stable or improved ambulation at approximately 2-year follow up [46].

### Psychiatric and cognitive manifestations

Psychiatric disturbances are often observed in patients with adolescent/adult-onset NP-C, and have been reported in up to 86% of cases, usually at initial presentation [25, 100]: they are relatively rare in patients with childhood-onset NP-C, although a few cases have been reported [101, 102]. Psychotic and mood-related symptoms are the most common psychiatric features reported in patients with NP-C (in 43–62% of patients) [26, 103–105]. Many adolescent/adult-onset patients (61–86%) exhibit cognitive decline, likely due to the fact that cognitive decline manifests in most patients of juvenile age onwards [27, 106].

Published evidence regarding the effects of miglustat on psychiatric manifestations is largely based on case reports and case series: changes in psychiatric status are not generally quantified using objective or systematic methods. Szakszon et al. reported complete recovery from psychosis after 1 year of miglustat therapy in a patient with juvenile-onset disease [102]. Maubert et al. described stabilised psychiatric symptoms and cognitive function, allowing cessation of antipsychotic therapy, in an adolescent/adult-onset patient after 1.7 years on miglustat [107]. Santos et al. reported improvements in indices for depression, affective and attention problems based on the child behaviour checklist (CBCL [108]), along with stabilisation/improvements in seizure control and movement, in a juvenile-onset patient [109]. Mattson et al. reported profound improvements in speech/language in a patient with adult-onset psychosis [110].

The mini-mental status examination (MMSE [60]) has been used to assess cognition in miglustat-treated patients in various studies. Small improvements in cognition were reported in miglustat-treated adolescent/adult-onset patients compared with those on standard care in the 12-month RCT [56]. In a retrospective, observational evaluation in a cohort in 21 adult-onset NP-C patients, initial improvement or stabilisation of cognitive function, as measured using the MMSE and FAB (frontal assessment battery), was reported during an average of 19 months of miglustat treatment [111]. MMSE analyses have indicated improvements in cognitive function over varying treatment periods (1–6 years) in individual case studies [52, 53, 105]. Developmental delay and cognitive impairment have also been assessed in selected paediatric patients in the Italian NP-C cohort using formal psychometric tests including Griffith’s mental developmental scale and Wechsler-Bellevue scales (WPPSI, WISCR, WAISR) [48, 112].

### General clinical impairment/disability evaluations

A number of NP-C disability scales that quantify overall ‘functional disability’ in NP-C based on categorical assessments of core neurological domains have been formulated to establish easy-to-use, focussed clinical tools suitable for physicians who are not experts in metabolic diseases (see Additional file 1: Table S1). The original NP-C disability scale was developed by Iturriaga et al. [24], and was later modified by Pineda et al. [45] to provide equal weighting for four core domain subscales (ambulation, manipulation, language and swallowing). Other forms of the original scale have followed similar assessment rationales, but have addressed additional neurological aspects. For instance, the scale developed by Fecarotta et al. for use in the Italian NP-C cohort included subscales for developmental delay/cognitive impairment, seizure activity and dystonia (as a separate domain), and was therefore better suited for assessing childhood-onset disease [48].

To date, NP-C disability scales have been used to evaluate effects of miglustat on patient impairment/disability in a large, retrospective observational cohort study [42], in the international NPC Registry [46], and in a number of national NP-C cohort studies [44, 45, 47, 48]. NP-C disability scales have also been measured in parallel with a number of putative neuropathological markers to achieve clinical validation (See *Neuropathological markers* section) [57, 67, 80].

In a retrospective observational cohort study, NP-C disability scores were reported for 66 patients (mean age 9.7 [0–32] years) treated with miglustat in clinical practice settings [42]. Overall neurological stabilisation was observed during a median 1.5 (0.1–4.5) years of treatment. While neurological disease was stabilised across all age-at-onset groups, the size of treatment effects was greater in patients with the juvenile- and adolescent/adult-onset forms than in those with early- or late-infantile onset. Prospective longitudinal NP-C disability scale assessments have been reported for 92 patients across all age-at-onset categories (range < 1–44.6 years) in the international NP-C Registry who received ≥1 year of continuous miglustat therapy [46]. Reduced annual disease progression was reported during a mean of 3.9 (1.1–9.8) years on therapy. In line with findings from the retrospective cohort study, the proportion of improved/stable patients increased across neurological onset categories in the following order: early-infantile (33%) < late-infantile (50%) < juvenile (79%) < adolescent/adult (94%).

Improved/stabilised neurological manifestations have consistently been reported based on prospective cohort studies in Italy [48, 113] and France [44], and case series reported in Spain [45] and Iran [47]. In the Italian national NP-C cohort, improved/stabilised disability was measured using a modified version of the Iturriaga scale over a median 5.9 (4.0–8.0) years of treatment in the majority (56%) of evaluable patients who started miglustat early on in the disease course (< 3.5 years after neurological onset) [48]. Notably, beneficial effects were much less frequent in patients who started treatment late on in the disease course. Patients with lower rates of neurological deterioration at treatment start responded better than those with higher rates of progression. In addition, developmental delay was stabilised or improved in the majority of patients (68%). A Spanish paediatric NP-C case series reported a relative lack of effect of miglustat on systemic symptoms such as splenomegaly [45]. However, neurological status generally appeared stabilized in juvenile-onset patients, while smaller neurological effects were observed in patients with early-infantile and late-infantile onset disease who were at a more advanced stage of disease treatment start. In an Iranian paediatric case series [47], a general stabilisation of scores was observed based on a disability scale designed specifically for use in Iranian patients, which incorporated ocular-motor function and seizure activity, along with improvements in psychomotor delay in early- and late-infantile onset patients.

A post-hoc analysis of data from the 12-month miglustat RCT evaluated disease stability based on subjective appraisals of all core clinical symptoms assessed during the trial (HSEM-α, swallowing, SAI, and MMSE) [114]. Among 29 patients who received ≥12 months of miglustat treatment, 21/29 (72%) were classified as having stable disease (i.e., no deterioration in swallowing, SAI *and* MMSE, *or* deterioration in HSEM-α only) [114]. These data were supported by similar multi-parameter analyses in the retrospective cohort analysis reported by Pineda et al. [42].

### Plasma biomarkers

Quantitative analysis of plasma oxysterols (e.g., cholestane-3β,5α,6β-triol [C-triol], 7-ketocholesterol [7-KC]), certain plasma bile acids (e.g., 3β,5α,6β-trihydroxycholanic acid), and certain lysosphingolipids (e.g., lyso-SM-509) have been shown to be highly effective in the diagnosis and screening of NP-C [12–17, 115, 116]. There are currently no data to support their use for disease or efficacy monitoring. Plasma levels of these markers have not been shown to correlate with neurological disease stage [14, 115, 116]. The presumed reason for this is that the main therapeutic action of miglustat is in the brain, whereas overall marker levels in blood reflect whole-body disease impact, contributed mostly by visceral impairment (e.g., hepatomegaly, splenomegaly). Previous published data indicate that miglustat has relatively little effect on visceral symptoms in NP-C [45].

### Treatment outcomes

Rates of disease progression and overall life expectancy vary greatly in NP-C and are highly influenced by age at onset of neurological symptoms. Estimates of age at onset and prognosis in US and European national NP-C cohorts have been reported previously [6]. While there are rare published cases where patients have survived into the sixth or even seventh decade of life or have never exhibited neurological manifestations [117, 118], almost all NP-C patients die prematurely.

Precise causes of death are not consistently reported in NP-C, but data from two separate patient cohorts indicate bronchopneumonia subsequent to food or fluid aspiration as the reason for early mortality in approximately 60% of patients [119, 120]. Dysphagia has been recorded in up to 80% of patients with NP-C [21–23], is by far the most common cause of aspiration pneumonia, and has been shown to represent a major risk factor for patient mortality [121].

In a meta-analysis of all available data from studies of miglustat treatment effects in NP-C, miglustat was reported to confer a potential survival benefit [121]. Comparison of untreated NP-C patients (*N* = 97) with those who received miglustat (*N* = 90; all age groups) revealed a vast numerical difference in the number of deaths over a 5-year period: 74 deaths among untreated patients versus three in the treated group. Kaplan-Maier estimates of survival indicated that this difference was statistically significant (*p* = 0.044).

## How to assess miglustat efficacy: Utility of disease monitoring methods

**Fig. 1:**
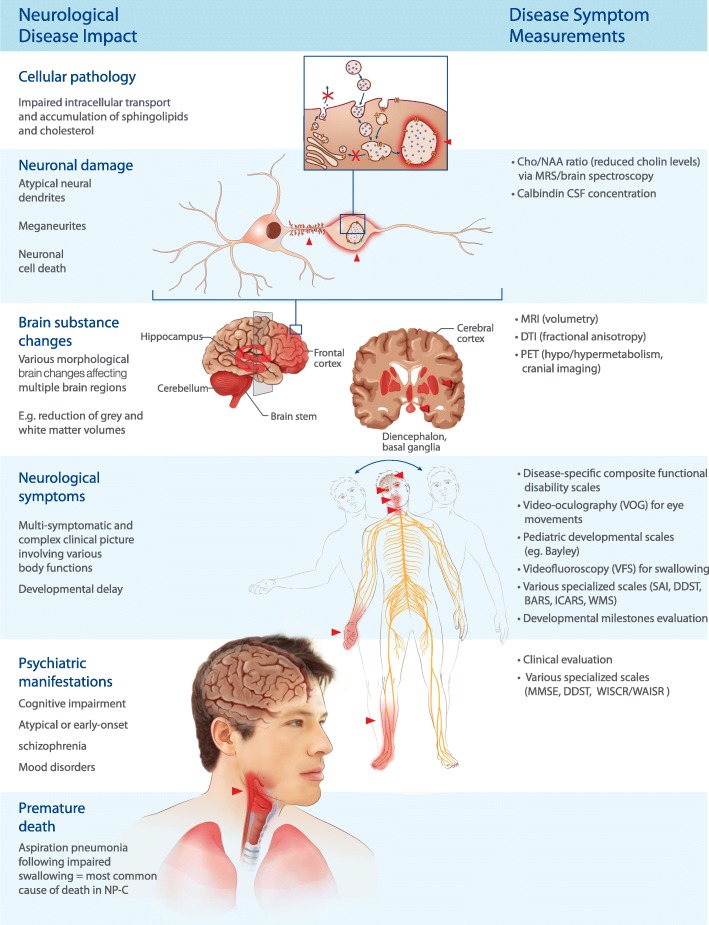
From biochemical and cellular/neuronal effects to clinical efficacy and improved outcomes

Figure 1 provides an overview of the relationship between NP-C cellular pathology and neuronal damage, brain substance changes, neurological symptoms, psychiatric manifestations and, ultimately premature death in NP-C, and lists some of the respective measurements that can be applied to track changes in each of these features. Several objective and subjective markers have been developed that can be used to assess each of the main neurological manifestations of NP-C, including general clinical impairment (NP-C disability scales), ambulation (SAI), ocular motor changes (HSEM-α, HSEM-β, HSG), swallowing (VFS, clinical grading), and imaging (e.g., MRS Cho/NAA ratio), and neurotransmission (TMS) (Table 4).

**Table 4:** Overview of NP-C disease markers

Type	Description and experience	Subjective/objective	Drawbacks	Diagnosis	Disease/treatment monitoring
General clinical impairment					
NP-C disability scales	• Categorical measures • Extensive use in clinical cohorts and case series	Subjective	• Variability of scales • Not very useful in EI pts	–	X
Developmental delay / cognitive impairment	• Subjective scales (MMSE, DDST) • Objective scales (WISCR/WAISR, Bayley-III) • Used in RCT, paediatric cohorts and case series/reports	Subjective/objective	• Limited disease specificity • Subjective measures	–	X
SAI	• General, practical scale • Used in key developmental clinical studies and case series	Subjective	• Limited disease specificity • Subjective measure	–	X
Ocular motor assessments (VOG)					
Saccadic eye movements (HSEM-α; HSEM-β, gain/latency)	• Well-characterised measure • Used in key developmental clinical studies and cohort studies	Objective	–	X	X
Swallowing assessments					
Clinical observation	• Multiple, varied assessments • Used in key developmental clinical studies	Subjective	• Time-consuming	–	X
VFS	• Direct measures standardised based on dysphagia scales • Used in multiple case series	Objective	• Not widely available	–	X
Neuroimaging					
MRI (volumetry)	• Extensive published information • Used in cohort studies/case series	Objective	• Non-specificity • Only late-stage changes seen	–	X
DTI (FA parameters)	• Sensitive measures • Used in a clinical cohort and a case report	Objective	• Non-specificity • Requires specific expertise	–	X
MRS (Cho/NAA ratio)	• Sensitive measures • Used in clinical cohorts	Objective	• Non-specificity • Requires specific expertise	–	X
PET (hypo/hyper-metabolism)	• Dynamic, functional measures • Used in a case series	Objective	• Non-specificity • Requires specific expertise	–	X
Neurotransmission					
TMS (cortical plasticity)	• Functional measure of neurological function • Used in a case series	Objective	• Non-specificity • Requires further validation	–	X
Biomarkers					
Filipin staining	• Previous ‘gold standard’ marker based on patient skin biopsies • Extensively published use	Objective	• Labour-intensive: difficult to perform and interpret • Expensive • Not widely available	X	–
Plasma ChT	• Widely available LSD marker • Extensive published use in LSDs including NP-C	Objective	• Non-specificity • Null-ChT mutations • Lack of correlation with clinical manifestations	–	X
CSF Cho/NAA ratio	• Used in a cohort study/case series	Objective	• Limited published experience • Invasive assay procedure	–	X
CSF calbindin	• Highly specific marker • Used in a cohort study	Objective	• Invasive sampling procedure (spinal tap)	–	X
Plasma oxysterols (c-triol, 7-keto)	• Rapid, cost-effective markers • Widely used in diagnosis	Objective	• Lack of published data from disease monitoring	X	–
Plasma bile acids	• Rapid, cost-effective markers • Highly practical (useable in DBS) • Limited published experience	Objective	• Lack of published data from disease monitoring	X	–
Lysosphingolipids (e.g., Lyso-SM-509)	• Rapid, cost-effective markers • Highly practical (useable in DBS) • Limited published experience	Objective	• Lack of published data from disease monitoring	X	–

*Bayley-III* Bayley scales of infant development [50], *ChT* chitotriosidase, *CSF* cerebrospinal fluid, *DBS* dried blood spots, *DDST* Denver developmental screening test [49], *DTI* diffusion tensor imaging, *EI* early-infantile, *FA* fractional anisotropy, *HSEM* horizontal eye movements, *MMSE* mini-mental status evaluation [60], *MRI* magnetic resonance imaging, *MRS* magnetic resonance spectroscopy, *PET* positron emission tomography, *SAI* standard ambulation index, *TMS* transcranial magnetic stimulation, *VFS* videofluoroscopic studies, *VOG* video-oculography, *WAISR* Wechsler Adult Intelligence Test, *WISCR* Wechsler Intelligence Scale for Children [112]

NP-C progresses slowly in most patients, and its clinical manifestations are highly variable from individual to individual. It would be highly desirable to use simple, widely available and reliable laboratory measurements to assess response to interventions. Such measurements are characterised as biomarkers or surrogate markers (see Table 5). A surrogate marker is defined as “…a laboratory measurement or physical sign that is used in therapeutic trials as a substitute for a clinically meaningful endpoint that is a direct measure of how a patient feels, functions, or survives and is expected to predict the effect of the therapy” [122]. In contrast, a biomarker is simply defined as “…a laboratory measurement that reflects the activity of a disease process” [123].

**Table 5:** Recommendations on NP-C marker selection for following disease development or treatment efficacy

• NP-C disability scale based clinical measures are easy to use and broadly acknowledged.	
• Simple, focussed NP-C disability scales (e.g. Pineda scale [45]) may be preferred over more wide-ranging measures (e.g., the NIH severity scale [90]).	
• The efficient application of an NP-C marker as part of a research study does not guarantee that the marker will be useful in individual patients in a hospital setting.	
• Imaging and laboratory marker methods should ideally be applied using established, locally available methods and expertise.	
• Specific imaging markers (e.g., MRI, DTI, VFS) provide objective, quantitative data, and can be applied independently of patient age.	
• The application of laboratory markers should be considered in relation to patients’ or carers’ acceptance and access.	
• Diagnostic NP-C biomarkers (oxysterols, lysosphingolipids, bile acids) do not currently qualify as effective methods for disease monitoring over time.	

Some methods, particularly those based on imaging (MRI, DTI, VFS), are widely accessible in hospital settings, provide objective, quantitative data, and can be applied independent of patient age. Visual analyses can be relatively non-specific for diagnostic purposes. However, they are highly specific for long-term monitoring as they can accurately and specifically capture longitudinal changes in affected brain regions over time. However, to be truly useful for disease monitoring in NP-C, these techniques require specialist evaluation (e.g., by a neuroradiologist) and interpretation. Likewise, specialised imaging methods such as PET and MRS are not readily available at all treatment centres as they require access to significant scanning hardware and expertise.

Other techniques (e.g., VOG) require a degree of cooperation that can be difficult to achieve in children and elderly patients, such as quantitative assessments of ocular motor impairment or evaluations of ambulation (in the SAI and NP-C disability scales). CSF calbindin assays, which require collection of CSF by lumbar puncture and quantification by immunoassay, require forward planning and necessary laboratory expertise, and are therefore more suited to research use in clinical studies.

NP-C disability scales have provided a great deal of valuable data on the clinical efficacy of miglustat. Despite being derived from subjective clinical observation, these scales are generally well suited for assessing treatment efficacy in clinical practice settings. The majority of studies assessing miglustat have been based on the original, simple scales reported by Iturriaga et al. in 2006 [24] and Pineda et al. in 2010 [45]. The more comprehensive neurological severity scale described by Yanjanin et al. [90], which is based on the US NIH NP-C cohort, has not been so widely used for longitudinal disease/efficacy monitoring in clinical practice. However, it has proved useful for assessing overall neurological disease severity in the clinical trial setting.

The original NP-C disability scale reported by Iturriaga et al. [24] and the modified version reported by Pineda et al. [45] have been applied most widely to monitor disease progression and treatment effects, but different scales including extra neurological domains have also been developed. As a result, certain clinical NP-C disability scales appear to be more relevant in early-infantile, late-infantile and juvenile-onset patients than in adolescent/adult patients. For instance, the scale applied by Fecarotta et al. in the Italian paediatric NP-C cohort included developmental delay/cognitive impairment, and may be an appropriate choice for assessing children aged > 6 years [48]. On the other hand, this scale does not take seizures or ocular movements into account. The Pineda et al. scale includes both of these domains as well as language delay [45].

## Clinical use of miglustat

Miglustat is indicated for the treatment of progressive neurological symptoms in both children and adults with NP-C [36]. The recommended dose for adolescent and adult patients is 200 mg t.i.d., and should be reduced in proportion to body surface area in paediatric patients, as per manufacturer’s instructions [36]. Based on expert consensus treatment should be initiated as soon as any neurological manifestations appear [5, 6, 36]. In patients who do not have neurological manifestations, but for whom there is a known family history and disease course, treatment can be commenced at the anticipated time of neurological onset [5, 124].

In general, miglustat therapy should be continued as long as patients continue to derive discernible therapeutic benefits with an acceptable tolerability and safety profile [5]. Treating physicians, patients and family members should be aware of what to expect from miglustat therapy, taking into account individual patient characteristics (e.g., age at onset, symptom severity). In general, based on clinical experience to date, it can take 6–12 months to observe clinical benefits in early-infantile onset cases and over 2 years in later-onset disease. Any decisions to alter or discontinue ongoing miglustat treatment should be taken with careful consideration of risk versus benefit and patient tolerability [5].

## Miglustat tolerability: Potential impact on effectiveness

As with any medication, the tolerability profile of miglustat in NP-C has the potential to affect clinical effectiveness. The most frequent adverse events (AEs) recorded in miglustat-treated patients during clinical trials and in clinical practice settings were mainly gastrointestinal (e.g., diarrhoea, flatulence and abdominal pain/discomfort) [36, 41, 43, 56, 125–127]. The gastrointestinal tolerability of miglustat at the start of treatment has the potential to affect patient compliance with therapy and needs to be monitored closely [127]. It is noteworthy that such effects can be minimised or even avoided using dietary modifications or, in some cases, careful up-titration of miglustat dosing at treatment start [36, 127]. Transient physiological tremor of the hands has been reported in over half of patients during the initial weeks of therapy, but usually resolves after the 1–3 months on treatment and can be helped by temporary dose reduction [36]. Weight loss has also been reported in both adults and children on miglustat, but has not been associated with any clinical sequelae and has either minimal or no effect on normal growth in paediatric patients [41]. Mild reductions in platelet counts that were not associated with bleeding have been observed in some NP-C patients during treatment with miglustat, and monitoring of platelet counts is recommended in patients with low platelets at start of treatment [36].

Peripheral neuropathy was reported as an AE in two patients with Gaucher disease (GD) who received miglustat during the initial GD registration trial [69]. While this prompted increased safety vigilance in miglustat-treated patients in subsequent years [125, 128, 129], peripheral neuropathy is a very rare complication in NP-C. A search of the literature reveals only a few published cases of this neurological manifestation in NP-C, all of which were in patients who did not receive miglustat [130–132].

## Conclusions

Since its initial approval in 2009, clinical experience with miglustat in the treatment of NP-C has increased markedly, as documented in numerous clinical cohort studies, case series and case reports. A weakness of the published literature is the scarcity of data from prospective, RCTs. RCTs are challenging to design, fund and execute in a rare, progressive disease such as NP-C, especially when no alternative disease-modifying therapies have been approved. Nevertheless, consistent with the clinical heterogeneity of NP-C, a wide variety of methods have been used to assess the effects of miglustat on a number of disease manifestations. Available parameters address changes in brain neurochemistry, metabolism, structure, CSF markers and neurotransmission, and changes in ocular-motor function, swallowing, movement, psychiatric manifestations, cognitive function and overall clinical disability.

Imaging studies based on DTI and MRS have demonstrated reduced levels of choline (a neurodegeneration marker), and Cho/NAA ratio (indicating increased neuronal viability) in the brain. Less reduction in fractional anisotropy (an axonal/myelin integrity marker) has also been observed in certain brain regions. In MRI studies, potential protective effects of miglustat on cerebellar and subcortical structures were shown to correlate with clinical symptom severity, although longitudinal MRI findings are subject to a high degree of variability. Observations of reduced CSF calbindin levels during treatment may represent a potential further means of tracking proposed protective effects of miglustat on cerebellar Purkinje cells. Observed changes in TMS parameters indicate roles for GABA-ergic, NMDA and cholinergic receptor-mediated neurotransmission in the effects of miglustat on dystonia, cognition and possibly, seizure activity.

Owing to the neurodegenerative nature of NP-C, disease stabilization is considered the best attainable therapeutic goal as irreversible damage/loss of neurones has likely already occurred in most patients by the time a diagnosis of NP-C is confirmed and targeted treatment initiated [6]. The main aims of targeted therapy are therefore to improve patient quality of life and maintain physical function.

Improved or stabilised core neurological manifestations have consistently been reported in miglustat cohort studies and case series based on NP-C disability scales that assess typical neurological symptoms. A range of these scales have been employed in studies reported to date. The more simple scales (e.g., those of Iturriaga et al. [24] and Pineda et al. [45]) are particularly useful in clinical practice and were used in the majority of studies covered in this review. The more comprehensive neurological severity scale developed based on the NIH NP-C cohort has been reported only in selected clinical trial settings to date [5, 90]. The range of disability scales that are available raises a difficulty, in that studies on treatment effects are not easily comparable with one-another and certainly do not allow statistical meta-analyses, which are important in rare diseases such as NP-C where patient numbers are limited. There is therefore an ongoing need for the development of a unified, validated disability scale that can be applied more widely across studies to measure treatment effects, certainly in patients aged ≥4 years.

Treatment benefits seem less predictable in patients with early-infantile-onset NP-C than in late-infantile-, juvenile- and adolescent/adult-onset groups. Therapeutic effects are also less pronounced in patients with severe manifestations at treatment start, and in those who start treatment late on in the course of disease. Objective, quantitative assessments indicate improvement/stabilisation of swallowing function (VFS) and ocular motor function (saccadic eye movement indices).

A published report has indicated beneficial effects of miglustat on prognosis in NP-C [121]. This may partly be due to slower progression of dysphagia, and a subsequent delay to disease stages where severe bronchopneumonia due to food or fluid aspiration becomes more likely. Further data are required to confirm this.

Overall, published data have shown that the treatment effects of miglustat in NP-C can be monitored on a variety of levels. In clinical practice physicians can choose from a range of accessible methods to monitor disease progression depending on individual case presentations, prevalent symptoms pertinent to specific patient cohorts, and local/regional health infrastructure. Regardless of the measures employed, it should be borne in mind that NP-C is a progressive disease, and patients should be followed up at 6–12-monthly intervals to allow proper longitudinal assessment.

## Additional file

Additional file 1: **Table S1.** Comparison of disability scales. (DOCX 45 kb)

## Abbreviations

CBCL: child behaviour checklist
Cho: choline
CNS: central nervous system
Cr: creatine
CSF: cerebrospinal fluid
DTI: diffusion tensor imaging
FA: fractional anisotropy
GSLs: glycosphingolipids
HSEM-α: horizontal saccadic eye movement alpha
HSG: horizontal saccadic gain
LTP: long-term potentiation
MMSE: mini-mental status examination
MRI: magnetic resonance imaging
MRS: magnetic resonance spectroscopy
NAA: *N*-acetylaspartate
NFTs: neurofibrillary tangles
NP-C: Niemann-Pick disease Type C
*NPC1/NPC2*: mutations in NP-C gene 1 or NP-C gene 2
PET: positron emission tomography
RCT: randomised controlled trial
SAI: short-latency afferent inhibition
TMS: transcranial magnetic stimulation
VFS: videofluoroscopy
VOG: video-oculography
VSSP: vertical supranuclear saccade palsy
