# Supplementary material for: Miglustat in Niemann-Pick disease type C patients: a review
Source: Orphanet J Rare Dis. 2018 Aug 15;13:140. doi: 10.1186/s13023-018-0844-0 (PMC6094874; doi:10.1186/s13023-018-0844-0)
Supplement: Supplementary file 1 — Table S1. Comparison of disability scales. (DOCX 45 kb) [file 13023_2018_844_MOESM1_ESM.docx]

# Table S1 Comparison of disability scales

| **Disability score item** | **Severity/definition** | **Further details/wording variations** | **Scoring†** | | | | |
| --- | --- | --- | --- | --- | --- | --- | --- |
|  |  |  | **Iturriaga [24]** | **Pineda  [45]** | **Karimzadeh [47]** | **Fecarotta** **[48]** | **Yanjanin**  **[90]**** |
| **Ambulation*** | Normal | – | 1 | – | 1 | 0 | 0 |
|  | Clumsiness | 'Normal gait with minor loss of control' [47] | – | 1 | 2 | – | 1 |
|  | Autonomous ataxic gait | 'Ataxia' [47]; 'or not walking by 18 months of age' [90] | 2 | 2 | 3 | 0.33 | 2 |
|  | Outdoor assisted ambulation | – | 3 | 3 | – | – | – |
|  | Indoor assisted ambulation | 'Assisted ambulation' [48]; 'Assisted ambulation or not walking by 24 months of age' [90] | 4 | 4 | – | 0.67 | 4 |
|  | Wheelchair bound | 'Unable to walk' [47] | 5 | 5 | 4 | 1 | 5 |
| **Manipulation*** | Normal | 'Fine and gross motor skills: normal' [47] | 1 | – | 1 | – | 0 |
|  | Mild tremor | 'Tremor' [42] | – | 1 | – | – | – |
|  | Slight dysmetria / dystonia | Allows autonomous manipulation; 'Tremor while grasping objects and hand writing' [47]; 'independent manipulation' [90] | 2 | 2 | 2 | – | 1 |
|  | Mild dysmetria / dystonia | Require help for several tasks but able to feed themselves  Unable to write/grasp objects; 'requires little or no assistance' [90] | 3 | 3 | 3 | – | 2 |
|  | Moderate dysmetria / dystonia | 'Limited fine motor skills, difficulty feeding self' [90] | – | – | – | – | 4 |
|  | Severe dysmetria / dystonia | 'Gross motor limitation' [90]; requires assistance in all activities | 4 | 4 | – | – | 5 |
| **Dystonia^‡^** | Normal | Absence of abnormalities | – | – | – | 0 | – |
|  | Slight dystonia | Only with actions, not disabling/interfering with activities | – | – | – | 0.33 | – |
|  | Moderate dystonia | Noticeable on action of distal body parts or intermittently at rest  Interfering with normal activities, causing moderate impaired function | – | – | – | 0.67 | – |
|  | Severe dystonia | Noticeable at rest and/or causing complete impairment of function | – | – | – | 1 | – |
| **Dysmetria^‡^** | Normal | Absence of abnormalities | – | – | – | 0 | – |
|  | Mild | Mild difficulties reaching the nose or an object | – | – | – | 0.33 | – |
|  | Moderate | Moderate difficulties reaching the nose or an object | – | – | – | 0.67 | – |
|  | Severe | Prevents reaching nose or an object | – | – | – | 1 | – |
| **Language*** | Normal | – | 1 | – | 1 | 0 | 0 |
|  | Delayed acquisitions | – | – | 1 | – | – | – |
|  | Mild dysarthria | Understandable; 'easily understood' [90] | 2 | 2 | 2 | 0.33 | 1 |
|  | Moderate dysarthria | Poor comprehensive language [48] | – | – | – | 0.67 | – |
|  | Severe dysarthria | Only comprehensible to some members of the family  'Poor comprehensive language' [48] | 3 | 3 | 3 | – | 2 |
|  | Non-verbal communication | 'Anarthria' [48]; 'functional communication skills for needs' [90] | 4 | 4 | – | 1 | 3 |
|  | Absence of communication | 'Minimal communication' [90] | 5 | 5 | – | – | 5 |
| **Swallowing*^,‡^** | Normal | 'Absence of difficulty to swallow' [48] | 1 | – | 1 | 0 | 0 |
|  | Difficulty chewing (abnormal) | Long feeding times; 'Abnormal chewing' [45]; 'Mild dysphagia (only for solids)' [47]; 'cough while eating' [90] | – | 1 | 2 | 0.25 | 1 |
|  | Occasional dysphagia | 'Arching or stiffening of neck during feeding; "gurgly", hoarse, or breathy voice quality' [48]; 'moderate difficulty with solids and minor dysphagia for liquids' [47]; additive subsections for liquids and solids (each scoring +1) [90] | 2 | 2 | 3 | 0.50 | Up to +2 |
|  | Daily dysphagia | 'Excessive drooling or food/liquid coming out of the mouth or nose, coughing or gagging during meals, difficulty breast feeding' [48]; 'Severe dysphagia (difficulty for solids and moderate difficulty for liquids)' [47] | 3 | 3 | 4 | 0.75 | Up to +4 |
|  | NG tube or gastric tube for supplemental feeding | – | – | – | – | – | 4 |
|  | NG tube or gastric button feeding | 'Refusing liquid or food of different texture, NG tube or gastric button feeding' [48]; 'Unable to swallow' [47] | 4 | 4 | 5 | 1 | 5 |
| **Seizures** | Normal | 'No seizures' [47]; 'no history of seizures' [90] | – | – | Not assigned | – | 0 |
|  | History of single seizure | – | – | – | – | – | 1 |
|  | Occasional seizures | 'Rare seizures' [90] | – | 1 | – | – | 2 |
|  | Seizures controlled with antiepileptic drugs | – | – | 2 | Not assigned | – | 3 |
|  | Seizures resistant to antiepileptic drugs | 'Refractory seizures' [47]; 'seizures difficult to control with meds' [90] | – | 3 | Not assigned | – | 5 |
| **Ocular movements** | Normal, absence of abnormalities | – | – | – | – | – | 0 |
|  | Slow ocular pursuit | – | – | 1 | 1 | – | – |
|  | Mild vertical ophthalmoplegia | 'Detected by physician only' [90] | – | – | – | – | 1 |
|  | Vertical ophthalmoplegia | 'Vertical gaze palsy' [47]; 'functional VSGP' [90] | – | 2 | 2 | – | 2 |
|  | Total VSGP | 'Abnormal horizontal saccades may be present' [90] |  |  |  |  | 3 |
|  | Complete ophthalmoplegia | 'Vertical and horizontal gaze palsy' [47] | – | 3 | 3 | – | 5 |
| **Developmental delay / cognitive impairment^‡^** | Normal | Normal achievement of development milestones; self-sufficient without support; IQ >84 (absent intellectual disability) [48] | – | – | – | 0 | 0 |
|  | Mild psychomotor delay | Mild delay to development milestones; patient fairly self-sufficient with minimal support needs; communication skills; IQ 50–69 or 70–84 (borderline disability) [48]; 'mild learning delay, grade appropriate for age' [90] | – | – | – | 0.33 | 1 |
|  | Moderate psychomotor delay | Moderate delay to milestones; patient able in self-care tasks; limited/moderate support; some communication skills; IQ 35–49 [48]; 'moderate learning delay, individualised curriculum or modified work setting' [90] | – | – | – | 0.67 | 3 |
|  | Severe psychomotor delay | Severe delay milestones; patient completely unable in basic self-care skills; extensive support; completely unable in basic communication; IQ 20–34 (severe intellectual disability) or <20 (profound intellectual disability) [48]; 'severe delay plateaus, no longer in school or no longer able to work, some loss of cognitive function' [90] | – | – | – | 1 | 4 |
|  | Minimal cognitive function | – | – | – | – | – | 5 |
| **Hearing** | Normal | 'All tones ≤15 dB HL' [90] | – | – | Not assigned | – | 0 |
|  | Abnormal | – | – | – | Not assigned | – | – |
|  | High frequency hearing loss (PTA ≤15 dB HL, >15 dB HL in high frequencies) | – | – | – | – | – | 1 |
|  | Slight-mild hearing loss (PTA 16–44 dB HL) | – | – | – | – | – | 2 |
|  | Moderate hearing loss (PTA 45–70 dB HL) | – | – | – | – | – | 3 |
|  | Severe hearing loss (PTA 71–90 dB HL) | – | – | – | – | – | 4 |
|  | Profound hearing loss (PTA >90 dB HL) | – | – | – | – | – | 5 |
| **Memory** | Normal | – | – | – | – | – | 0 |
|  | Mild short-term or long-term memory loss | – | – | – | – | – | 1 |
|  | Moderate short-term or long-term memory loss (gets lost) | – | – | – | – | – | 2 |
|  | Difficulty following commands | – | – | – | – | – | 3 |
|  | Unable to follow commands or short- and long-term memory loss | – | – | – | – | – | 4 |
|  | Minimal memory | – | – | – | – | – | 5 |

**Severity/definition based on original Iturriaga scale [24]; **'Modifiers to this scale can add to overall neurological severity scores (gelastic cataplexy, narcolepsy, behaviour, psychiatric, hyperreflexia, incontinence, auditory brainstem response, respiratory) [90]; ^†^each domain rated on a five-point scale from normal (0 or 1) to worst (3, 4 or 5) except the Fecarotta scale which is rated from 0 (best) to 1 (worst) [48]; ^‡^severity/definition based on original Fecarotta scale [48]; dark grey shaded boxes = not covered; PTA, pure-tone average (reported on audiogram); '–' = severity category not included; 'not assigned' = domain assessed but no quantitative score assigned; NG, nasogastric tube.*
